# Morphokinetic analysis of early human embryonic development and its relationship to endometriosis resection: a retrospective time-lapse study using the KIDScore™ D3 and D5 implantation data algorithm

**DOI:** 10.1007/s00404-023-07008-6

**Published:** 2023-05-14

**Authors:** Saskia-Laureen Herbert, Claudia Staib, Theresa Wallner, Sanja Löb, Carolin Curtaz, Michael Schwab, Achim Wöckel, Sebastian Häusler

**Affiliations:** Department of Obstetrics and Gynaecology, University Medical Centre Würzburg, Josef-Schneider-Straße 4, 97080 Würzburg, Germany

**Keywords:** Endometriosis, Embryo quality, Time-lapse technology, KIDScore™, Complete resection

## Abstract

**Research question:**

Does complete resection of endometriosis improve embryo quality as assessed by morphokinetic parameters using time-lapse microscopy?

**Design:**

For this retrospective study we analysed 237 fertilised, cultured and transferred embryos from 128 fresh IVF and/ or ICSI transfer cycles. Endometriosis was confirmed or excluded by laparoscopy. Patients were stimulated with recombinant FSH using GnRH agonist and antagonist protocols. After fertilisation, a time-lapse incubation system was used for observation. Embryo quality was assessed using the KIDScore™ D3 and D5 implantation data algorithm.

**Results:**

The analysis showed a median KIDScore™ D5 of 2.6 (on a scale of 1 to 9.9) for embryos from patients with endometriosis without complete resection. The control group without endometriosis achieved a score of 6.8 (*p* = 0.003). The median score for embryos from endometriosis patients with complete resection was 7.2, which was a significant increase compared to embryos from patients without complete resection (*p* = 0.002). We observed an effect size of *r* = 0.4 for complete resection versus no resection of endometriosis using the KIDScore™ D5. There were no differences in KIDScore™ D3 between the three patient groups. Pregnancy and miscarriage rates showed the same clinical trends. In three of our four case series of patients who underwent IVF/ ICSI cycles before and after complete resection, we found a marked improvement in embryo quality after complete resection.

**Conclusions:**

Complete resection of endometriosis could significantly improve the otherwise poor embryo quality of patients undergoing IVF-procedures. The data, therefore, strongly support recommending surgery to patients with endometriosis prior to assisted reproduction.

## What does this study add to the clinical work

**Table:** 

In this study we analysed embryos from patients with and without endometriosis undergoing IVF/ICSI using time-lapse imaging. Our data showed that complete resection of endometriosis has a positive effect on early embryo morphokinetic parameters. Surgical intervention prior to IVF or ICSI may therefore be an appropriate way to treat endometriosis patients suffering from infertility.	

## Introduction

Endometriosis is one of the most common gynaecological conditions. It is characterised by the presence of cells similar to those in the endometrium, but located outside the uterine cavity. Approximately 4–30% of women of childbearing age are affected by endometriosis [10, 38, 41]. The prevalence is higher in women with infertility, ranging from 20 to 50% [11, 27].

As endometriosis is a heterogeneous chronic disease, there are a number of different presentations. Dysmenorrhea is the most common symptom. Endometriosis is further associated with infertility and miscarriage [34]. Monthly fertility is lower [20], as is the live-birth rate [9]. The causes of this disease affecting fertility are multifactorial. There are mechanical, molecular, genetic, and environmental aspects, but a definitive explanation is still controversial [25]. In vitro fertilisation (IVF) studies have shown that women with more advanced endometriosis have poor ovarian reserve, poor oocyte and embryo quality and poor implantation rates [8, 29]. Altered immune function and endocrine milieu can be found in patients with endometriosis [39, 44]. Mechanical disorders such as adhesions alter the pelvic anatomy. This is associated with impaired oocyte release and retrieval, myometrial contraction and embryo transport [19]. Luteal phase disruption leads to reduced endometrial receptivity [28]. Ovulation, oocyte production, fallopian tube function and sperm quality are negatively affected by increased levels of inflammatory cells and cytokines [28]. Inflammatory peritoneal fluid shows a toxic effect on embryos [28]. This may affect morphokinetic timing, but there is only limited data on morphokinetics and endometriosis [15, 36].

Treatment for endometriosis-related infertility varies depending on the stage of endometriosis, sperm factors and the patient’s age. Options include surgery, medical treatment and assisted reproductive technology (ART). Currently, IVF is considered to be the most effective treatment for patients with endometriosis, low ovarian reserve and or patients who are older than 35 years [14]. Although IVF does not alter the biology of endometriosis, data show a similar birth/pregnancy rates in patients with and without endometriosis undergoing IVF-ET (embryo transfer) [12]. In general, medical treatment should not be favoured in patients with endometriosis-related subfertility, as there is no convincing evidence to support the benefit of progesterone treatment before trying to conceive, but there are conflicting data for IVF. Luteal phase GnRHa (gonadotropin-releasing hormone agonist) downregulation followed by IVF/ ICSI (intracytoplasmic sperm injection) results in similar pregnancy rates in women with endometriosis compared to women with tubal factor infertility [30]. Surgery can restore the pelvic anatomy, remove endometriotic implants and should therefore be able to reduce the negative effects of endometriosis on embryonic development.

In view of the above, the question arises as to whether complete resection of the endometriotic tissue can improve embryo quality.

In recent years, time-lapse microscopy has become increasingly popular in embryo incubation. It allows undisturbed embryo culture and morphological assessment without removing the embryos from the incubator. With constant monitoring during cultivation, a time-lapse technology allows the identification and mapping of all embryo-related morphological events as they occur, known as morphokinetics [13]. Time-lapse imaging has introduced a new era of scoring tools to help identify the best embryo for transfer. Several algorithms have been developed to correlate morphokinetic variables with embryo implantation potential.

The implantation data algorithm KIDScore™ D3 is a model based on six annotations from more than 3300 embryos shared by 24 centres [32] and is generally applicable regardless of the culture conditions in different ART laboratories and the fertilisation method. It allows the identification of slow or fast-developing embryos, irregular cleavage and embryos that are not developing optimally. The KIDScore™ D5 is based on developmental information from approximately 5200 embryos with known implantation status at day 5 embryo transfer [42].

The aim of this study was to evaluate the quality of embryos transferred in different study groups, comparing patients with endometriosis confirmed by endoscopy, patients with surgically removed endometriosis and patients with no evidence of endometriosis on diagnostic endoscopy.

## Materials and methods

### Study design

237 embryos (endometriosis group: *n* = 126; non-endometriosis control group: *n* = 111) undergoing infertility treatment at our clinic from 2014 until 2017 were included in this retrospective study. The time interval was chosen as the same core teams for performing the surgery and the assisted reproductive therapies were responsible in this period. Inclusion criteria were female patients aged between 18 and 45 years undergoing IVF and/ or ICSI treatment. The endometriosis group was divided into two subgroups. It was differentiated between embryos from patients with endometriosis that underwent complete resection (d3 *n* = 37; d5 *n* = 36) and embryos from patients with endometriosis that did not underwent complete resection (d3 *n* = 15; d5 *n* = 20). If this information was missing, we excluded these embryos.

Endometriosis was confirmed laparoscopically with histological examination of a biopsy. When patients were scheduled for complete resection of their endometriosis the surgery could be performed laparoscopically in all cases. The primary trocar was inserted subumbilically after insufflating the abdominal cavity with CO_2_ via a Veress needle. Further 5 mm-trocars were placed in the lower belly appropriate for reaching the individually observed endometriotic lesions. After systematic evaluation of the whole abdominal situs a stage was determined. “Complete resection” was defined as the complete removal of every endoscopically visible endometriotic lesion but did not include systematic excision of an eventual adenomyosis uteri. Describing the stage of endometriosis rASRM (reversed American Society for Reproductive Medicine) score was used [5]. This score was developed by the reversed American Society for Reproductive Medicine. A common score for endometriosis. The rASRM is a scoring system. The score is using points corresponding the size of peritoneal and ovarian endometriosis. The points are also assigned for adhesions. The sum of the points results in a score of four grades.

The in vitro culture was performed in a closed time-lapse incubator (EmbryoScope^®^, Vitrolife) up to day 3 or up to day 5.

In addition, we provide a small case series of four patients. They underwent IVF/ ICSI before and after complete endometriosis resection.

Ethical approval was obtained from the Ethics Committee of Würzburg University (file number 20191007 01).

### Controlled ovarian stimulation oocyte retrieval, fertilization, embryo analysis, embryo transfer, pregnancy assessment and endometriosis surgery

Ovarian hyperstimulation was performed according to the agonist or antagonist protocol as previously described e. g. by Diedrich et al. [17]. Oocytes were aspirated by transvaginal follicle aspiration 36 h after an ovulation induction with 5000 or 10,000 IU human chorionic gonadotropin (HCG, Predalon^®^ MSD). Aspiration was performed by experienced surgeons specialised in reproduction. In case of endometriosis the aspirate did not have any contact to endometrioma. If necessary, aspiration system was changed. Cumulus-oocyte complexes were collected in SynVitro Flush media (Cooper Surgical, Ref. 15,760,125) at 37 °C and then transferred into Continous Single Culture medium (CSC-C, Irvine Scientific, Ref. 90,165) at 37 °C and 6.8% CO_2_. The embryo cultures were covered with oil for embryo culture (Irvine Scientific, Ref. 9305). For IVF oocytes were inseminated with a progressively motile sperm concentration ranging between 0.1 and 0.5 × 10^6^/ml in CSC. Prior to the ICSI procedure cumulus cells were removed from the oocytes with hyaluronidase enzyme (Cumulase^®^, Cooper Surgical, Ref. 16,125,000) and denuding pipettes with appropriate lumen sizes (175–135 µm, Vitromed, Jena, Germany, Ref. V-Den-135, − 150, − 175). Introcytoplasmatic sperm injection was performed in multipurpose handling medium (MHM, Irvine Scientific, Ref. 90,166) and polyvinylpyrolidone (PVP, Cooper Surgical, Ref. 10,905,000). After ICSI the oocytes were cultured in CSC-C medium in an Embryo Slide culture dish (Vitrolife, Ref. FR-S-ES-D) for 3 to 5 days without media change. In the case of IVF, fertilized oocytes were transferred to the Embryo Slide culture dishes on day 1 after fertilization check. Embryos were cultured in an EmbryoScope (Vitrolife) at 6.8% CO_2_ and 5% O_2_ at 37 °C. During embryo culture embryo evaluation was constantly performed according to Instanbul consensus [4]. Briefly, embryo quality was calculated in terms of number of blastomeres, cell fragmentation and symmetry. For the evaluation of KIDScore™ D3 and D5 only the best embryos, which were choosen for transfer or cryopreservation, according to the embryologist’s view were annotated on D3 or D5, depending on the day of embryo transfer. KIDScore analysis was performed using the Embryoviewer software (Vitrolife) and evaluated by applying KIDScore™ D3 (continuous scale from 1 to 5) or KIDScore™ D5 (continuous scale running from 1 to 9.9) according to the day of embryo transfer. Using the morphokinetic KIDScore™ D3 the model assigns a low score to those with the statistically lowest probability of implantation and a higher score to those with a statistically higher probability of implantation. KIDScore™ D5 considers morphology and morphokinetic traits. The higher the score the higher the chance of implantation.

KIDScore™ D3 is based on six annotations: the number of pronuclei equals 2 (2PN), time from insemination to pronuclei fading(tPNf), time from insemination to the 2-cell stage (t2), time from insemination to the 3-cell stage (t3), time from insemination to the 5-cell stage (t5), time from insemination to the 8-cell stage (t8). KIDScore™ D5 is based on 8 annotations: 2PN, t2, t3, t4, t5, timing of full blastocyst (tB), quality of Inner Cell Mass (ICM) (a, b, c) and the quality of the Trophectoderm (a, b, c).

Pregnancy was assessed using vaginal ultrasound. All patients with positive pregnancy test at home get an appointment for ultrasound in the 7th pregnancy week. Life birth rate is defined as number of deliveries per fresh embryo transfer. Pregnancy rate is defined as number of pregnancies per fresh embryo transfer. Abortion rate is defined as number of abortions out of all pregnancies.

The University medical Centre Würzburg is an endometriosis centre level III. Surgery is performed by specialized surgeons. Patients obtained complete resection. Not all hospitals fulfil the qualifications for adequate treatment of endometriosis. Therefore, endometriosis centres were established. In this process, the hospitals are audited and certified by an independent body, EuroEndoCert. This institution uses strict guidelines for certification. There are three stages of centres: endometriosis centre (level I), clinical endometriosis centre (level II), clinical and scientific endometriosis centre (level III) (all: [35]).

### Statistical analysis

**Fig. 1:**
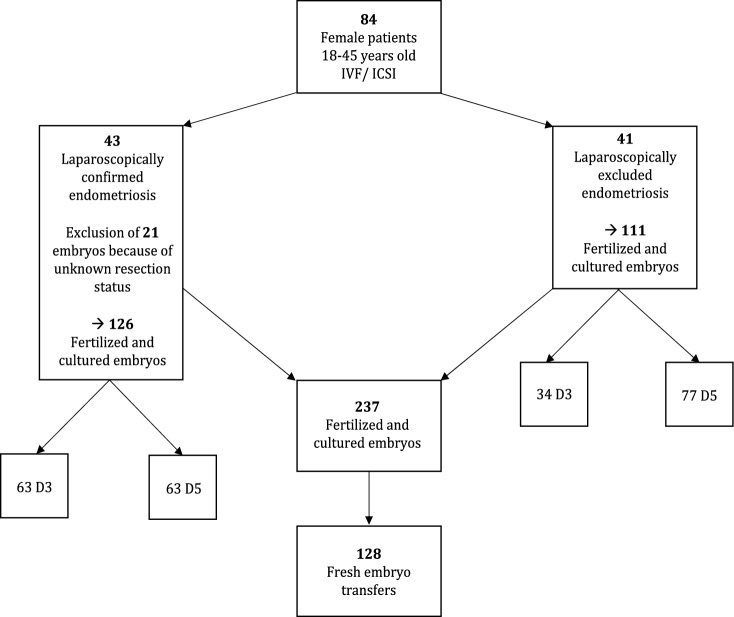
Flow chart of choosing patients and embryos. 237 embryos from 84 patients undergoing IVF/ ICSI were included. 43 patients with 126 embryos had laparoscopically confirmed endometriosis. 41 patients with 111 embryos did not have endometriosis. KIDScore™ D3 was used for 63 embryos of the endometriosis group and for 34 embryos of the control group. KIDScore™ D5 was used für 63 embryos of the endometriosis group and for 77 of the control group. 128 fresh embryo transfers were performed

The data handling and the statistical operations were performed using Microsoft Excel 2019 (Microsoft, USA) and SPSS 25 (IBM, USA). For non-parametric comparisons of the ordinally scaled KIDScore ™ measurements the Mann–Whitney *U*-test was used to check for statistical significance. Fisher’s exact test was performed to determine the shown significance levels of the pregnancy rate comparisons. The Computation of the biological effect size “*r*” according to Cohen was performed as described by Fritz et al., 2012 the computed test statistic of the mentioned statistical test in SPSS and the respective case number. *p*-values < 0.05 were considered statistically significant (Fig. 1).

## Results

### Sample–description

In this study, we enrolled 84 patients, 43 with histologically proven endometriosis and 41 without endometriosis (control group, laparoscopically confirmed). In total we had data from 261 embryos. We analysed 237 cultured embryos, 126 from patients with endometriosis and 111 from patients without endometriosis. A total of 128 single or double embryo transfers were performed. For 97 embryos we annotated a D3 score, and for 140 embryos we annotated a D5 score. The difference is statistically significant. The embryos were assigned to one of the four groups depending on the day of transfer and on the confirmation of endometriosis. Age, BMI, stimulation cycles, oocyte retrieval and transferred embryos per cycle of these four groups are displayed in Table 1.

**Table 1:** Characteristics D3 & D5

	Endometriosis D3	Control group D3	Endometriosis D5	Control group D5
Patients’ characteristics				
Age (years)	35.1 (34.1, 36.0)	35.6 (34.5, 36.6)	34.9 (33.7, 36.1)	33.8 (32.9, 34.8)
BMI (kg/m^2^)	24.6 (23.4, 25.9)	24.9 (23.3, 26.6)	22.5 (21.8, 23.2)	25.1 (24.0, 26.1)
Clinical data				
Total number of cycles	37	20	31	40
Retrieved oocytes per cycle	8.1	8.8	13.4	12.2
Cultivated oocytes per cycle	3,1	3,1	4,7	4.0
Transferred embryos per cycle	1.8	1.8	2.1	2.0
Life birth rate per cycle	0.2	0.2	0.2	0.2
Number of IVF/ICSI cycles	15/22	8/12	7/24	11/29
Number of previous cycles with ovarian stimulation (IUI, IVF, ICSI)	107	104	106	0.93
Agonist/Antagonist stimulation	17/20	8/12	12/19	15/25
Tubal factor	9	8	8	7
Endometriosis	37	0	31	0
PCO	0	1	0	6
Male factor in %	44	43	44	66

Values are means (95% CI) or absolute values

We observed a significant difference concerning body mass index (BMI) of endometriosis group D5 and control group D5 (*p* = 0.005) (Table 1). In addition, significantly fewer oocytes were aspirated per cycle in endometriosis group D3 than in endometriosis group D5 and control group D5 (*p* = 0.001, *p* = 0.009, respectively) and fewer embryos were transferred per cycle than in endometriosis group D5 (*p* = 0.028). The rate of live births per performed IVF/ICSI cycle showed no significant difference in the four groups (Table 1).

### KIDScore™ day 3 and day 5–differences between patients with and without endometriosis

**Fig. 2:**
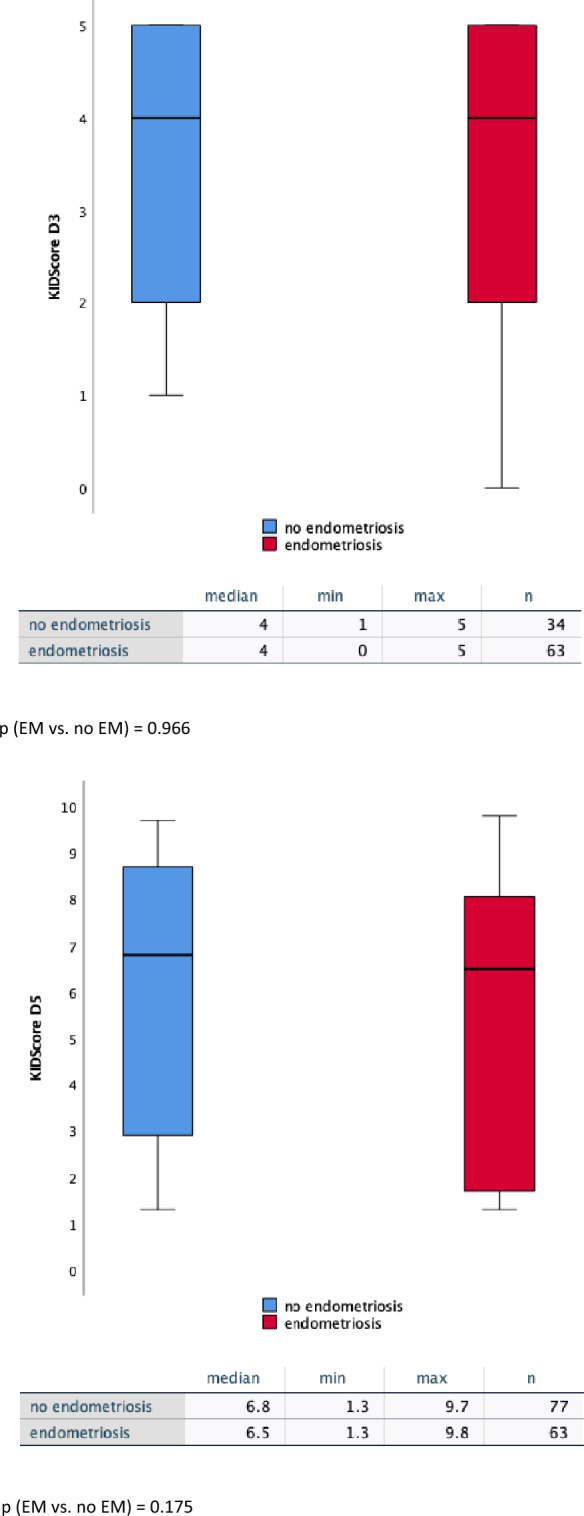
Effect of diagnosed endometriosis on KIDScore™. KIDScore™ D3 (left), KIDScore™ D5 (right)—comparison of embryo quality between patients with endometriosis and without endometriosis via boxplot. Median (prominent line), absolute minimum (lower end of the boxplot), absolute maximum (higher end of the boxplot) and number of embryos are included in the table below. Computation of the test statistic as given in the figure was performed using the Mann–Whitney *U* test

Figure 2 shows the distribution of KIDScore™ measured on day 3 and day 5 for embryos from patients with and without endometriosis (regardless of whether the endometriosis was completely resected or not). On day 3 the median for endometriosis patients was 4 (on a scale from 1 to 5) as well as the median for non-endometriosis patients (control group). However, on day 5 a trend towards a lower embryo quality measured by time lapse monitoring was observed between the mentioned groups (*p* = 0.175): embryos from patients without endometriosis showed a higher KIDScore™ D5 (median 6.8) (on a scale from 1 to 9.9) than embryos from patients with endometriosis (median 6.5).

### KIDScore™ day 3 and day 5–impact of resection on embryo quality

**Fig. 3:**
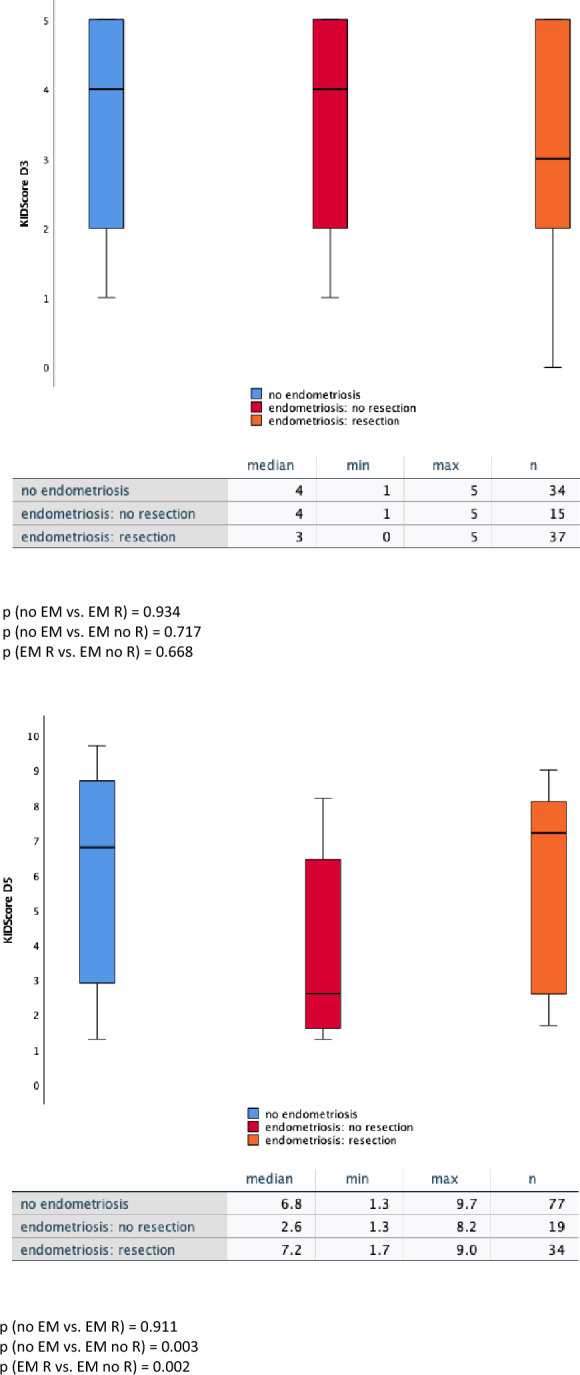
Effect of complete resection of endometriosis on KIDScore™. Shown is the comparison of the embryo quality measured by KIDScore D3 (left) and KIDScore d5 (right) between patients without endometriosis (“no endometriosis”), patients with resection of endometriosis (“endometriosis: complete resection”) and without resection of endometriosis (“endometriosis: no resection”). The presented boxplots show the values for absolute Minimum—Quartil—Median (prominent line)- Quartil–absolute Maximum) for each patient group. The absolute KIDScore values for median, minimum (“min”), maximum (“max”) as well es the number of embryos (“*n*”) are enlisted in the table below each chart. Computation of the test statistic as given in the figure was performed using the Mann–Whitney *U* test. (*p < 0.05, **p < 0.01)

Figure 3 also covers KIDScore™ day 3 and KIDScore™ day 5, but here the endometriosis group was divided into two subgroups. One consisted of embryos from patients with endometriosis who underwent complete resection of their endometric lesions. The other group contained only embryos from patients with histologically confirmed endometriosis and not being treated by complete resection. Of note, for analyses of day 3 11 embryos had to be excluded from further computations as it was not clearly documented that the complete surgery was performed as described in material and methods; for day 5 10 embryos had to be excluded. For KIDScore™ day 3 no significant differences in the embryo quality could be observed. In contrast to that KIDScore™ D5 showed a statistical highly significant difference: the median KIDScore™ D5 of embryos from patients without complete resection of their endometriosis was 2.6. In comparison to the control group which showed a median of 6.8 a significant decrease in embryo quality was recorded (*p* = 0.003). When the endometriosis was completely removed surgically, a median score of 7.2 was achieved. This indicates a highly significant increase (*p* = 0.002) in embryo quality compared to the KIDScore™ D5 patients without complete removal and is not differing significantly from the not-endometriosis bearing control group (*p* = 0.911). Computation of the biological effect size according to Cohen showed a moderate up to strong biological effect (*r* = 0.4) for endometriosis complete resection vs. no resection and a moderate biological effect (*r* = 0.3) for no endometriosis vs. endometriosis without resection.

### Pregnancy rate and abortion rate

Interestingly, complete resection also had a small impact on pregnancy and abortion rate.

Patients that underwent complete resection showed a similar pregnancy rate (32.6%) compared to patients without endometriosis (32.8%), (*p* = 1.000). Patients with endometriosis and no resection showed a trend towards a lower pregnancy rate (17.6%) compared to patients that underwent complete resection (*p* = 0.346) and also compared to patients without endometriosis (*p* = 0.370). Statistical difference was not observed.

After complete resection, the abortion rate was 44.4%. In contrast, in case of endometriosis without resection—although only two patients met this criterion—the abortion rate was 100%. The difference did not reach statistical significance (*p* = 0.455). The abortion rate in the group of women without endometriosis (42.9%) compared to women with non-resected endometriosis (*p* = 0.217) and compared to women with resected endometriosis (*p* = 1.000) did not differ significantly from each other.

All: (Fig. 4).

**Fig. 4:**
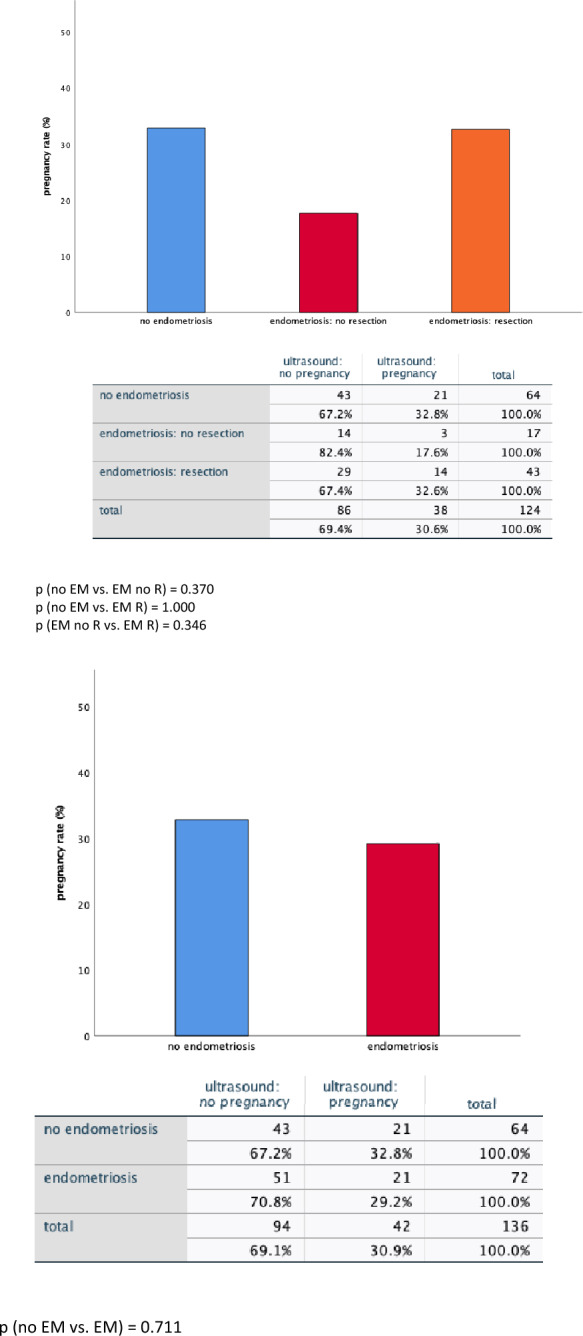
Effect of complete resection of endometriosis on pregnancy (**A**) and abortion (**B**) rate Shown is a comparison of pregnancy rate (**A**) and abortion rate (**B**) between patients without endometriosis and ones with endometriosis (left), respectively, patients without endometriosis, with resection of endometriosis and without resection of endometriosis (right). The given *p*-value was computed using the Fisher-Exact-test

### Case report—KIDScore™ day 3 and day 5 – impact of surgery on embryo quality

**Fig. 5:**
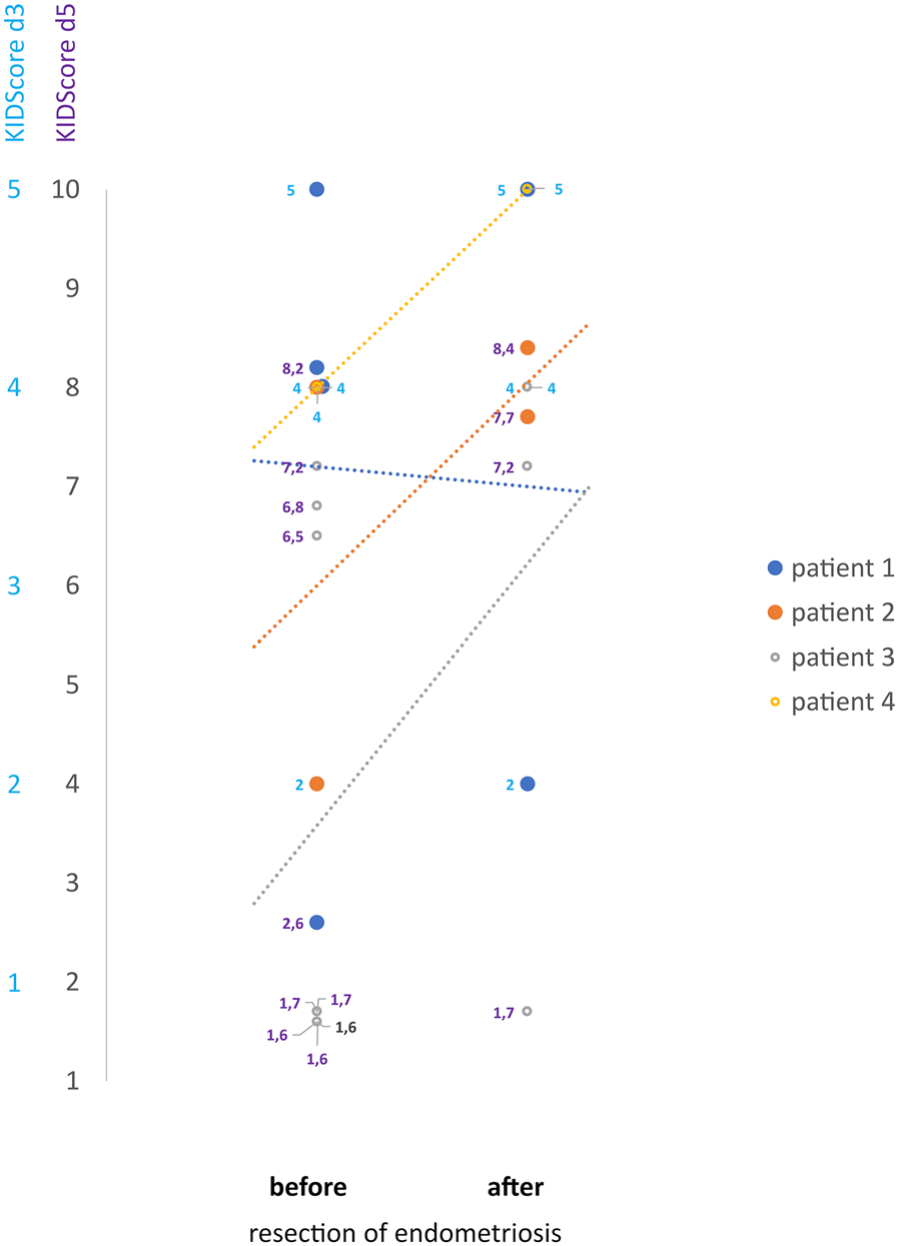
Case series–KIDScore™ in four patients undergoing IVF–surgery-IVF-treatment. All patients had IVF-treatments before and after a complete resection of their endometriosis. Forpatient 1 (blue), patient 2 (orange), patient 3 (grey), patient 4 (yellow) the respective KIDScores™ from each transferred embryo are depicted as colored dot before (left) and after (right) complete resection of endometriosis. The particular values for KIDScores™ D3 are inserted in light blue resp., for KIDScore™ D5 in purple for each embryo/dot. Additionally, a linear trendline illustrating the course of the KIDScores™ from before to after resection of the endometriosis is depicted in the diagram for each patient in matching colors (dotted line)

Figure 5 shows a small case series of four patients suffering from endometriosis and undergoing IVF-treatment before and after the complete resection of their endometriosis.

Patient 1 was 41 years old when she underwent her first ICSI treatment. Her BMI was 21.3 kg/m^2^. She had her first ICSI in March 2014 and was stimulated under protection of a GnRH analogon. Seven oocytes were retrieved, and two embryos were transferred. KIDScore™ day 3 was 4 and 5. There was no pregnancy achieved in this cycle. She started her next cycle in August 2014 with the same protocol. 11 oocytes were aspirated, two embryos were transferred. KIDScore™ day 5 was 8.2 and 2.6. ß-HCG was negative again. In November 2014 she underwent laparoscopy. Endometriosis rASRM II was diagnosed. Lesions were resected completely. Afterwards she had another ICSI in October 2015 stimulated with GnRH antagonist protocol. 5 oocytes were aspirated, 2 embryos were transferred. KIDScore™ day 3 was 5 and 2. Unfortunately, ß-HCG was negative again.

Patient 2 was 32 years old when she underwent her first ART treatment. Her BMI was 20.9 kg/m^2^. She already had her first diagnostic surgery because of endometriosis in 2004. From 2012 to 2014 she underwent many cycles of IVF and ICSI. The first ART in which the embryos were evaluated with time-lapse monitoring was in April 2014. She was stimulated with GnRH agonist protocol, 15 oocytes were aspirated, and 2 embryos transferred. KIDScore™ day 3 was 2 and 4. ß-HCG was negative. In September 2014 she underwent complete resection of endometriosis rASRM II. Two months later she went for another ICSI cycle, stimulated with GnRH antagonist protocol. 8 oocytes were aspirated, and 2 embryos transferred. KIDScore™ day 5 was 8.4 and 7.7. ß-HCG was positive, and she delivered one child.

Patient 3 was 31 years old when she started ART at our hospital. She already has been pregnant but interrupted the pregnancy because of a trisomia. Her BMI was 25.3 kg/m^2^. She did not lose weight through the years of treatment. She underwent diagnostic surgery because of endometriosis in March 2014. In May 2014 she had an ICSI, stimulated with GnRH agonist protocol. 12 oocytes were retrieved, and 2 embryos transferred. KIDScore™ day 5 was 7.2 and 6.8. ß-HCG was positive. Unfortunately, she had a spontaneous abortion. In November 2014 she started her next ICSI with the same stimulation protocol. 14 oocytes were aspirated, 2 embryos transferred, KIDScore™ day 5 was 1.6 for both embryos but ß-HCG was negative. The following ICSI-treatment was in February 2015. With the use of the same protocol for stimulation again 14 oocytes were retrieved, and 2 embryos transferred. KIDScore™ day 5 was 1.7 and 1.6, ß-HCG was negative again. In July 2015 she had another ICSI. With GnRH agonist protocol 9 oocytes were retrieved, and 2 embryos transferred. KIDScore™ day 5 was 1.7 and 6.5. ß-HCG was positive, but pregnancy ended in a spontaneous abortion. In September 2015 she underwent complete resection of deep infiltrating endometriosis ENZIAN A3 B2 C1. Shortly afterwards she started a new cycle of ICSI, but unfortunately we do not have the KIDScore™ of the two transferred embryos. The following ICSI treatment was in September 2016. 7 oocytes were retrieved, 2 embryos transferred, KIDScore™ day 3 was 4 for both embryos. ß-HCG was negative. She had another ICSI in December 2017. 8 oocytes were aspirated, 2 embryos transferred, KIDScore™ day 5 was 7.2 and 1.7 and ß-HCG negative.

Patient 4 was 39 years old when she started her first ICSI treatment. Her BMI was 24.2 kg/m^2^. In August 2014 she was stimulated with GnRH agonist protocol, only 5 oocytes were retrieved, KIDScore™ day 3 was 4 and ß-HCG was negative. In October 2014 she underwent complete resection of endometriosis rASRM II. Afterwards she was stimulated with the ultralong protocol in January 2015. Again only 5 oocytes were retrieved, KIDScore™ day 3 was 5 and ß-HCG was negative.

Patient 2, 3 and 4 showed an increasing KIDScore™/ increasing embryo quality after complete resection, whereas patient 1 showed a decreasing KIDScore™/ embryo quality after complete resection (Fig. 5).

## Discussion

In this study we analysed the effect of complete resection of endometriosis on embryo quality as measured by the KIDScore™ D3 and D5 algorithms. For some of the embryos from patients with endometriosis who underwent complete resection, we showed a statistically significant improvement in quality compared to embryos from patients with endometriosis and no resection using the D5 algorithm. The quality of embryos from patients with endometriosis who underwent complete resection was comparable to that of embryos from patients without endometriosis. A moderate to strong biological effect could be achieved by complete resection of endometriosis.

Interestingly, no study has compared the effect of complete endometriosis resection on embryo quality using the KIDScore™ D3 or D5 algorithm. Most studies on the effect of endometriosis resection on fertility only include implantation rate or live-birth rate, but do not include morphokinetic effects.

Since endometriosis has a profound effect on oocyte and embryo quality, selecting the embryo with potential for implantation is of great interest. In the past, there have been several scoring systems for early human development, all aimed at identifying the best embryo. The older scoring criteria [18, 37, 45] typically assessed static developmental time points using conventional microscopy. The introduction of time-lapse microscopy gave the opportunity to monitor developmental and morphokinetic changes of the embryo in real time. Using this technique, many predictive algorithms have been developed by many different laboratories, all with relatively small databases. The development of KIDScore™ D3 has given a universal algorithm that we can use to objectively analyse our data.

Although it is still controversial whether surgery improves fertility in endometriosis patients, there is evidence to support the use of laparoscopic surgery to improve fertility [6, 40]. The aim of surgery is to completely remove endometriotic lesions, divide periovarial and peritubular adhesions and restore physiological anatomy. Data from a meta-analysis showed an improvement in fertility and live-birth rate after laparoscopic surgery. This benefit has been demonstrated mainly in patients with minimal to mild endometriosis (all: [21]). Adamson and Pasta showed a higher pregnancy rate in patients with minimal endometriosis after laparoscopy and laparotomy than after medical treatment of subfertility [1]. A 10–25% increase in pregnancy rate has been observed in endometriosis patients following surgery [24].

The explanation for the improvement in subfertility in endometriosis with surgery may be multifactorial, depending on the pathogenesis. In addition to anatomical advantages, complete resection of endometriosis may minimize the inflammatory environment. This may explain the increase in embryo quality/KIDScore™ as inflammatory cytokines are toxic to embryos [28]. Unfortunately, there is a lack of data on moderate to severe endometriosis. In addition, randomised controlled trials are missing to determine the efficacy of surgical treatment of moderate to severe endometriosis regarding infertility.

Our data show the importance of complete resection in patients with endometriosis undergoing ICSI in terms of embryo quality as measured by KIDScore™ on day 5. This indicates that complete resection of endometriosis lesions may improve the quality of embryos from patients with endometriosis. We were not able to show an improvement in KIDScore for day 3 embryos. This finding is consistent with another similar study. Sanchez et al. were unable to demonstrate an effect of endometriosis on the in vitro development of D3 embryos, but did observe a reduction in ongoing pregnancy rates with endometriosis [33]. They have suggested that this may be due to altered endometrial receptivity, mainly due to inflammatory-related changes in gene expression, or a possible progesterone resistance in women with endometriosis [31].

The general morphokinetic algorithm for day 3 is based on six annotations and ranks the embryo into five groups, whereas the algorithm for day 5 is based on eight annotations and provides a ranking between 1 and 9.9. Our hypothesis is that the embryo quality assessment on day 3 is not as accurate as the embryo assessment on day 5. Some very important time-points, for embryo evaluation are only possible within an embryo culture of 5 days. This is consistent with other studies [22, 43] which suggest that significant differences in morphokinetic parameters in women with advanced age do not change before T8 (time to cleavage into 8 cells [43]. Another study [22] showed that tB (time to full blastocyst) is an important factor in assessing blastocyst quality. These findings show that adverse effects, such as endometriosis or age, may affect later time points in the development of the early embryo.

Freis et al. were able to show altered embryo morphokinetics in patients with endometriosis compared to non-endometriosis patients, resulting in poorer embryo quality [15, 16]. Consistent with our data, they found that it was not the timing of the specific events during early embryo development, but the relative kinetics of the embryo development that changed between the two study groups, with a negative effect for the endometriosis group.

Our case series showed a trend towards improvement in embryo quality within the case series. Three out of four patients showed an improvement in embryo quality after complete resection of endometriosis. When comparing the characteristics of the patients, there are two differences that may have affected the results. Patient 1 is the oldest, and it is established that the quality of oocytes and embryos declines with age [23, 34]. Another interesting difference is the time between complete resection and resumption of ART. Relapse cannot be excluded [7]. In line with this observation is another study [2], in which significantly higher ongoing pregnancy rates were observed in women with endometriosis with a short interval between surgery and IVF treatment compared with women with a longer interval between surgery and an IVF treatment cycle. Patient 1 had the longest interval without any reproductive treatment, as she had a break of 11 months after surgery. Patient 2 had a break of 2 months, patient 3 and patient 4 both had a break of 3 months.

We would like to mention some limitations of our study that should be taken into account when evaluating its results. First, this was a retrospective study and retrospective studies are controversial [26]. As data already exist, the impact of missing data is low. Acquisition of the control group can also be considered critical as it is not a random selection and statistical bias can occur. Another limitation is the fact that we cannot correlate the KIDScore™ with the implantation rate when two embryos are transferred. After transfer, it is not possible to differentiate the fate of the transferred embryos in respect of implantation. The fact that we included 128 cycles from 84 patients may also introduce bias. Better or worse embryo quality depends also on the patient/ couple themselves. The fact that only transferred embryos were scored with KIDScore™ may also be a limitation. It would have been interesting to know whether complete resection of endometriosis improves embryo quality in general or only in a subset of embryos.

A limitation is that the details of the algorithm of the KIDScore™ D5 have not been disclosed by the manufacturer, which prevents further endometriosis-specific conclusions. It should also be noted that the KIDScore™ D3 and D5 are designed to provide information on the implantation potential of the embryo, not for the live-birth rate (LBR). Although the assessment of embryo quality by time-lapse monitoring is considered an excellent tool that provides a lot of information about the embryos, the Cochrane review of time-lapse monitoring failed to find a clear distinction between LBR derived from embryos selected by time-lapse monitoring and conventional static morphological assessment [3]. As there are no significant differences in LBR within our study groups, the role of morphokinetics alone in predicting embryo performance should be questioned. However, additional studies are needed to look more closely at morphokinetics, the implantation and the ongoing pregnancy in endometriosis patients.

As infertility is common in patients with endometriosis, affecting approximately 20% of women of reproductive age, improving the management of endometriosis in terms of fertility is of great interest. The pathogenesis of endometriosis and endometriosis-related infertility is multifactorial and still not fully understood. This makes causal treatment difficult. However, knowing that endometriotic lesions produce inflammatory cytokines that are toxic to oocytes and embryos, resection of the lesions to reduce inflammation seems a plausible approach. Restoring the anatomy may also be an advantage of resection.

Our study showed a significant improvement in embryo quality (KIDScore™ D5) in patients with endometriosis after complete resection compared to patients without resection. However, as this was the first study to compare embryo quality with the KIDScore™ in relation to endometriosis resection, further studies are needed to confirm these results.

## Data Availability

The datasets generated during and/or analysed during the current study are available from the corresponding author on reasonable request.
